# The relation between dietary quality and healthy eating index with bone mineral density in osteoporosis: a case-control study

**DOI:** 10.1186/s12891-023-06704-3

**Published:** 2023-07-18

**Authors:** Marzieh Ghadiri, Mitra Soltani, Milad Rajabzadeh-Dehkordi, Shirin Gerami, Zainab Shateri, Mehran Nouri, Bahram Pourghassem Gargari

**Affiliations:** 1 Student Research Committee, Department of Biochemistry and Diet Therapy, Faculty of Nutrition and Food Sciences, Tabriz University of Medical Sciences, Tabriz, Iran; 2 Department of Clinical Nutrition, School of Nutritional Sciences and Dietetics, Tehran University of Medical Sciences (TUMS), Tehran, Iran; 3 Department of Community Nutrition, School of Nutrition and Food Sciences, Shiraz University of Medical Sciences, Shiraz, Iran; 4 Nutrition Research Center, School of Nutrition and Food Sciences, Shiraz University of Medical Sciences, Shiraz, Iran; 5 Department of Nutrition and Biochemistry, School of Medicine, Ilam University of Medical Sciences, Ilam, Iran; 6 Health Policy Research Center, Institute of Health, Shiraz University of Medical Sciences, Shiraz, Iran; 7 Nutrition Research Center, Department of Biochemistry and Diet Therapy, Faculty of Nutrition and Food Sciences, Tabriz University of Medical Sciences, Tabriz, Iran

**Keywords:** Healthy eating index, Dietary quality index, Bone density, Osteoporosis

## Abstract

**Background:**

In this study, we aimed to illustrate the association between the Healthy Eating Index (HEI) and Dietary Quality Index (DQI) with bone mineral density (BMD) among postmenopausal Iranian women with osteoporosis compared to the healthy control.

**Methods:**

In the current case-control study, 131 postmenopausal women with osteoporosis and 131 healthy postmenopausal women participated. Dual-energy X-ray absorptiometry was used to assess the lumbar vertebrae and femoral neck BMD. The subjects completed a validated food frequency questionnaire (FFQ), and then HEI and DQI were calculated based on the FFQ data. Crude and adjusted multivariable logistic regression was used to assess the relation between HEI and DQI with the odds of the femoral and lumbar BMD.

**Results:**

According to the results, participants in the last tertile of HEI were more likely to have higher femoral and lumbar BMD in the crude model (odds ratio (OR) = 0.38; 95% confidence interval (CI): 0.20–0.71 and OR = 0.20; 95% CI: 0.10–0.40, respectively) and also in the adjusted model (OR = 0.40; 95% CI: 0.20–0.78 and OR = 0.20; 95% CI: 0.10–0.41, respectively). Also, in terms of DQI-I, participants in the last tertile were more likely to have higher femoral and lumbar BMD in the crude model (OR = 0.23; 95% CI: 0.12–0.45 and OR = 0.29; 95% CI: 0.15–0.55, respectively) and also in the adjusted model (OR = 0.29; 95% CI: 0.14–0.58 and OR = 0.34; 95% CI: 0.17–0.67, respectively).

**Conclusions:**

The results of the current study supported the hypothesis that high-quality diets with healthy patterns can be clinically effective in maintaining bone health. Thus, recommendations regarding the consumption of nutrient-rich food groups in a healthy diet can serve as a practical non-pharmacological strategy against osteoporosis.

## Introduction

Bones change lifelong through the remodeling process to maintain structural integrity and regulate the balance of calcium and phosphorous [1]. This process happens according to osteoblasts’ and osteoclasts’ activities in formation and resorption, respectively. The imbalance between bone formation and resorption can lead to bone diseases such as osteoporosis and osteopenia [2]. Osteopenia is a condition in which a decrease in bone mineral density (BMD) and subsequent fractures due to fragility is seen [3]. The World Health Organization (WHO) has defined osteopenia as a T-score of BMD between − 1 to -2.5, while values less than − 2.5 are considered osteoporosis [4]. The T-score is the difference between the BMD of the patient and the normal young population divided by the standard deviation (SD) of the normal young population [5].

Osteopenia and osteoporosis can influence both genders, but postmenopausal women are more prone. Moreover, a history of bone fracture, older ages, and vitamin D and calcium deficiency are remarkable associated risk factors for osteopenia and osteoporosis [3, 6]. Other critical pathogenic mechanisms comprise unfavorable development and strength, bone loss due to extreme resorption and inappropriate structure, impaired compensatory activities to bone loss, and estrogen deficiency [7].

The Healthy Eating Index (HEI) is a measure to evaluate the nutritional quality of a diet based on the recommendations of the Dietary Guidelines for Americans [8]. This 13-component index considers multidimensional food groups regarding adequacy and moderation [9]. The Diet Quality Index (DQI) is another nutritional assessment that can evaluate diet variety, adequacy, moderation, and balance [10]. The DQI was constructed due to the importance of diet-associated chronic disease and undernutrition problems [11]. These dietary quality indices are inversely related to the risk of chronic diseases, including obesity, cancer, cardiovascular diseases, type 2 diabetes, and all-cause mortality [12–14].

Bone extracellular tissue consists of organic matrix and inorganic salts. While inorganic components include calcium, magnesium, phosphorous, sodium, potassium, zinc, and other ions, the organic part is composed of proteins, particularly collagenous proteins [2]. Thus, dietary factors from micronutrients (minerals and vitamins) to macronutrients and varied types of diets can positively or negatively affect bone health through changes in bone structure and metabolism, modification of paracrine and endocrine pathways, alteration in the homeostasis of bone compounds, and suppression of inflammatory processes [15–17]. Despite inconsistent observations, it’s claimed that high-quality healthy diets can serve as a protective approach against bone disease, mainly osteopenia, and osteoporosis [18]. To our knowledge, few studies have investigated the correlation between HEI and DQI with BMD. Thus, in the current study, we aimed to illustrate the association between HEI and DQI with BMD among postmenopausal women with osteopenia/osteoporosis compared with the healthy postmenopausal control.

## Materials and methods

### Study population

**Fig. 1:**
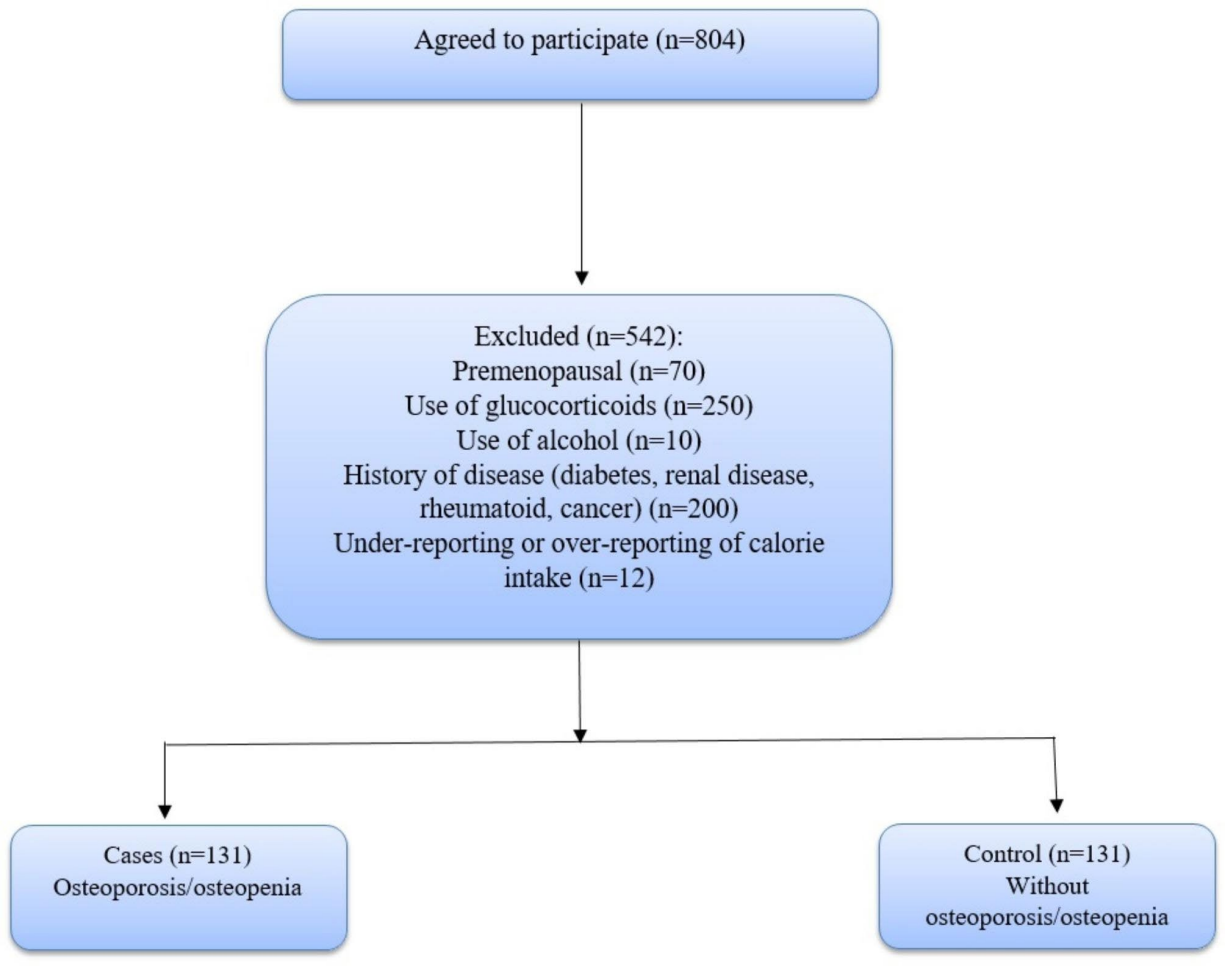
The flow chart of the study

In the current case-control study, 131 postmenopausal women with osteopenia/osteoporosis and 131 healthy postmenopausal women participated. These individuals were chosen from the Isfahan bone density measurement center in Iran from May to December 2021. The lack of a menstrual cycle in 12 months was considered menopause. In this study, the exclusion criteria were taking glucocorticoids (each dose for more than three months was excluded), consuming any kind of alcohol, premenopausal, diabetes, cancer, renal disease, and history of chemotherapy (Fig. 1). The present study’s details have been previously published [19, 20].

A general information questionnaire was used to gather information, such as drug use, smoking, and socio-demographic variables. Stadiometer was used to measure height, and a digital scale was used to measure body weight. Body mass index (BMI) was calculated as weight [21] divided by height squared (m^2^) [21].

The participant’s physical activity level was evaluated by the International Physical Activity Questionnaire (IPAQ) [22]. Women were divided into three groups based on the metabolic equivalent of task (MET)-minutes less than 600 MET-minutes/week: low activity, between 600 and 3000 MET-minutes/week: moderate activity, and more than 3000 MET-minutes/week: intense activity.

### Bone mineral density measurement

The method of dual-energy X-ray absorptiometry (DXA) was used for assessing the BMD of lumbar vertebrae and femoral neck (Horizon Wi (S/N 200,451)). The bone mass status was evaluated with WHO criteria (T-score more than − 1: normal BMD, T-score between − 1 and − 2.5: osteopenia, and T-score equal to or less than − 2.5: osteoporosis) [23].

### Dietary assessment and food grouping

A validated food frequency questionnaire (FFQ) was completed by individuals [24]. Also, we used HEI-2015 in our study [8, 25]. So, scores were calculated by 13 food groups. The maximum score was 100. The groups contain four components of moderation (added sugars, refined grains, saturated fats, and sodium) and nine components of adequacy (greens and beans, whole grains, whole fruits, total fruits, vegetables, protein foods, sea foods, dairy, and fatty acids (polyunsaturated fatty acid (PUFA) + monounsaturated fatty acid (MUFA)/saturated fatty acid (SFA)). The score of moderation components was between 0 and 10. The minimum and maximum range of adequacy components were 0 to 5, respectively. The score of every participant was calculated, and they were placed into tertiles.

DQI- International (DQI-I) contains four dietary components. First is food variety, with a score of 0 to 20 points. The food variety includes two elements, a wide variety of food categories (meats and meat products, fish, pulse products, fruits, grains, eggs, vegetables, milk, and milk products) and a within-group variety for protein foods (fish, meat products, milk products, eggs, pulse products). Second is adequacy (protein, grains, fruits, vegetables, fiber, calcium, vitamin C, ferric) and it scores between 0 and 40. The third is moderation (empty calorie foods, sodium, cholesterol, saturated fat, and total fat), with a score from 0 to 30 points. The fourth is overall balance (fatty acid and macronutrient ratios), with a score between 0 and 10. Finally, DQI-I scores from 0 to 100 [11, 26].

### Statistical analysis

For statistical analysis, SPSS (version 26) was used. P-value < 0.05 was considered statistically significant. For continuous variables, the mean with SD was used. For categorical variables, we used frequency and percentage. Independent samples T-test and chi-square test were used for continuous and categorical variables, respectively. Analysis of variance (ANOVA) test was used for the association between nutrient and food group intake across HEI and DQI tertiles. Crude and adjusted multivariable logistic regression was used to assess the relation between HEI and DQI with the odds of the femoral and lumbar abnormality. In the adjusted model, we controlled the effects of BMI, age, income, physical activity, education, taking vitamin D, and calcium supplements.

## Results

As Table 1 shows, the mean age of the control group was significantly lower than the case group (P = 0.036). The femoral and lumbar BMD was higher in the control group (P < 0.001 for both). Also, physical activity (P = 0.01), education level (P < 0.001), and vitamin D supplement (P = 0.018) were significantly different between the two groups.

**Table 1:** Baseline characteristics of study participants

Variables	Control (n = 131)	Case (n = 131)	P-value
Age (year)	56.47 ± 5.91	57.95 ± 5.42	**0.036**
BMI (kg/m^2^)	29.13 ± 3.31	29.78 ± 3.99	0.150
BMD femoral	0.78 ± 0.07	0.64 ± 0.09	**<0.001**
BMD lumbar	1.00 ± 0.08	0.81 ± 0.09	**<0.001**
Income Average (%) High (%)	53 (40.5) 78 (59.5)	65 (49.6) 66 (50.4)	0.086
Physical activity (%) Low Moderate	109 (83.2) 22 (16.8)	122 (93.1) 9 (6.9)	**0.01**
Education level (%) Under diploma Diploma Higher diploma	65 (49.6) 52 (39.7) 14 (10.7)	98 (74.8) 25 (19.1) 8 (6.1)	**<0.001**
Calcium Supplement (%) Yes No	32 (24.4) 99 (75.6)	32 (24.4) 99 (75.6)	0.557
Vitamin D Supplement (%) Yes No	76 (58.0) 55 (42.0)	58 (44.3) 73 (55.7)	**0.018**

Values have been presented as mean ± SD for continuous and frequency (percentage) for categorical variables.

Using independent samples T-test for continuous and chi-square test for categorical variables.

Table 2 shows the nutrient intake of participants. Protein and fiber were higher in HEI’s last tertile than in the first tertile (P < 0.001 for both). Also, vitamins A, B_6_, C, calcium, magnesium, iron, zinc, and copper were higher in the last tertile of HEI in comparison to the first tertile (P < 0.05 for all), but vitamin B_12_ was more in the second tertile of HEI (P = 0.007). Energy, carbohydrate, protein, and fiber were more in the last tertile of DQI-I (P < 0.001 for all). Moreover, vitamins A, E, B_6_, C, B_9_, calcium, magnesium, iron, zinc, and copper were higher in the last tertile of DQI-I compared to the first tertile (P < 0.05 for all). Sodium was more in the first tertile of both HEI and DQI-I (P < 0.05).

**Table 2:** Nutrient intakes between tertiles of HEI and DQI-I

	HEI				DQI-I			
Variables	T_1_ (n = 87)	T_2_ (n = 93)	T_3_ (n = 82)	P-value	T_1_ (n = 84)	T_2_ (n = 90)	T_3_ (n = 88)	P-value
Energy (kcal/d)	2098.02 ± 366.32	2131.07 ± 364.35	2153.00 ± 347.84	0.606	1990.80 ± 300.01	2150.22 ± 347.95	2233.14 ± 384.17	**<0.001**
Carbohydrate (g/day)	312.46 ± 56.32	314.47 ± 53.59	316.46 ± 47.70	0.886	289.40 ± 42.48	315.75 ± 50.09	336.95 ± 53.76	**<0.001**
Protein (g/day)	61.35 ± 11.49	67.99 ± 1.49	72.51 ± 13.74	**<0.001**	60.59 ± 12.01	68.42 ± 11.87	72.26 ± 12.48	**<0.001**
Fat (g/day)	73.27 ± 14.58	74.25 ± 14.55	74.97 ± 14.14	0.743	72.47 ± 13.13	75.44 ± 14.47	74.43 ± 15.45	0.388
Fiber (g/day)	27.12 ± 4.27	30.51 ± 4.14	35.93 ± 6.25	**<0.001**	27.64 ± 4.52	30.62 ± 4.69	34.83 ± 6.55	**<0.001**
SFA (g/day)	18.06 ± 5.08	19.12 ± 5.13	18.95 ± 4.44	0.310	17.54 ± 5.05	19.39 ± 5.30	19.15 ± 4.17	**0.027**
MUFA (g/day)	25.81 ± 4.37	26.88 ± 4.30	27.58 ± 5.57	0.053	26.22 ± 4.20	27.09 ± 4.69	26.89 ± 5.38	0.459
PUFA (g/day)	18.39 ± 3.23	18.96 ± 3.62	19.60 ± 4.06	0.101	18.63 ± 3.34	18.81 ± 3.20	19.44 ± 4.33	0.310
Vitamin A (RAE/day)	352.55 ± 173.92	468.82 ± 171.99	643.76 ± 357.46	**<0.001**	310.02 ± 125.89	466.69 ± 175.85	670.64 ± 331.97	**<0.001**
Vitamin E (mg/day)	21.46 ± 4.90	22.32 ± 3.72	22.52 ± 4.92	0.265	21.31 ± 4.54	21.61 ± 4.23	23.35 ± 4.60	**0.006**
Vitamin B_6_ (mg/day)	1.51 ± 0.24	1.69 ± 0.30	1.88 ± 0.41	**<0.001**	1.45 ± 0.25	1.69 ± 0.30	1.92 ± 0.35	**<0.001**
Vitamin B_9_ (µg/day)	461.81 ± 74.03	462.38 ± 75.75	467.86 ± 92.10	0.865	425.16 ± 74.33	460.76 ± 68.62	504.12 ± 78.88	**<0.001**
Vitamin B_12_ (µg/day)	2.49 ± 1.40	3.10 ± 1.50	3.06 ± 1.29	**0.007**	2.40 ± 1.29	3.21 ± 1.74	3.02 ± 1.01	**<0.001**
Vitamin C (mg/day)	101.28 ± 48.05	141.08 ± 52.82	189.45 ± 78.89	**<0.001**	94.15 ± 44.26	133.76 ± 43.23	199.08 ± 73.83	**<0.001**
Sodium (mg/day)	3842.22 ± 558.24	3627.39 ± 543.95	3584.57 ± 459.17	**0.003**	3799.17 ± 488.14	3698.86 ± 510.73	3562.81 ± 577.02	**0.014**
Calcium (mg/day)	342.91 ± 240.58	504.86 ± 279.11	626.17 ± 312.83	**<0.001**	372.47 ± 299.79	499.89 ± 288.69	589.24 ± 275.05	**<0.001**
Magnesium (mg/day)	379.56 ± 56.48	415.31 ± 69.25	456.73 ± 76.14	**<0.001**	386.26 ± 69.05	414.89 ± 63.04	446.72 ± 78.04	**<0.001**
Iron (mg/day)	14.82 ± 2.04	15.04 ± 2.02	15.68 ± 2.39	**0.027**	14.29 ± 1.81	15.21 ± 1.81	15.96 ± 2.50	**<0.001**
Zinc (mg/day)	10.13 ± 2.08	11.15 ± 2.15	11.80 ± 2.42	**<0.001**	10.10 ± 2.31	11.12 ± 1.99	11.77 ± 2.35	**<0.001**
Copper (mg/day)	1.46 ± 0.24	1.58 ± 0.27	1.70 ± 0.29	**<0.001**	1.43 ± 0.22	1.58 ± 0.25	1.71 ± 0.29	**<0.001**

HEI, Healthy Eating Index; DQI-I, Dietary Quality Index-International; SFA, saturated fatty acid; PUFA, polyunsaturated fatty acid; MUFA, monounsaturated fatty acid; RAE, retinol activity equivalents

Values have been shown as mean ± SD.

Using one-way ANOVA.

According to Table 3, whole grains, fruits, vegetables, nuts, legumes, and dairy were higher in the last tertile of HEI compared to the first tertile (P < 0.05 for all). Refined grains, sweets and sugar beverages, and processed meat were more in the first tertile (P < 0.05 for all). In terms of DQI-I, fruits, vegetables, legumes, dairy, and meats were higher in the last tertile in comparison to the first tertile (P < 0.05 for all) but the whole grains group was higher in the first tertile of DQI-I (P = 0.001).

**Table 3:** Food group intakes among tertiles of HEI and DQI-I

	HEI				DQI-I			
Variables	T_1_ (n = 87)	T_2_ (n = 93)	T_3_ (n = 82)	P-value	T_1_ (n = 84)	T_2_ (n = 90)	T_3_ (n = 88)	P-value
Whole Grains (g/day)	204.12 ± 38.56	208.28 ± 48.14	227.44 ± 51.60	**0.003**	224.72 ± 46.04	216.11 ± 43.50	198.31 ± 48.78	**0.001**
Fruits (g/day)	320.15 ± 152.36	453.07 ± 159.01	591.75 ± 204.94	**<0.001**	304.72 ± 150.55	431.50 ± 140.43	614.54 ± 186.22	**<0.001**
Vegetables (g/day)	171.59 ± 86.66	225.15 ± 74.40	318.09 ± 133.53	**<0.001**	167.81 ± 71.47	218.87 ± 78.10	319.96 ± 132.17	**<0.001**
Nuts (g/day)	6.88 ± 1.37	11.13 ± 1.22	12.40 ± 1.37	**0.010**	8.23 ± 1.55	9.52 ± 0.98	12.54 ± 1.41	0.067
Legumes (g/day)	20.23 ± 10.08	27.14 ± 13.00	32.96 ± 17.44	**<0.001**	20.98 ± 10.57	26.44 ± 12.35	32.34 ± 17.75	**<0.001**
Oil (g/day)	29.33 ± 5.00	29.60 ± 4.92	29.15 ± 6.93	0.879	30.15 ± 4.83	29.54 ± 5.64	28.45 ± 7.06	0.163
Refined Grains (g/day)	295.80 ± 89.60	244.28 ± 83.18	163.82 ± 75.19	**<0.001**	224.49 ± 110.19	239.94 ± 93.08	243.56 ± 91.80	0.406
Dairy (g/day)	177.07 ± 135.58	269.39 ± 165.80	295.79 ± 165.52	**<0.001**	205.76 ± 176.49	257.87 ± 167.63	275.24 ± 137.95	**0.015**
Meats (g/day)	36.17 ± 14.90	38.27 ± 12.55	38.39 ± 13.23	0.482	30.94 ± 13.03	39.89 ± 13.08	41.64 ± 12.26	**<0.001**
Sweets & Sugar Beverages (g/day)	27.67 ± 4.72	22.31 ± 4.69	5.86 ± 1.37	**0.001**	23.57 ± 4.64	21.60 ± 4.82	11.82 ± 2.45	0.099
Processed Meat (g/day)	48.97 ± 17.73	32.04 ± 17.73	24.91 ± 16.91	**<0.001**	36.97 ± 22.46	37.60 ± 34.31	31.73 ± 24.18	0.304

HEI, Healthy Eating Index; DQI-I, Dietary Quality Index-International.

Values have been shown as mean ± SD.

Using one-way ANOVA.

Based on Table 4, participants in the last tertile of HEI were more likely to have higher femoral and lumbar BMD in the crude model (odds ratio (OR) = 0.38; 95% confidence interval (CI): 0.20–0.71 and OR = 0.20; 95% CI: 0.10–0.40, respectively) and also in adjusted model (OR = 0.40; 95% CI: 0.20–0.78 and OR = 0.20; 95% CI: 0.10–0.41, respectively). In terms of DQI-I, participants in the last tertile were more likely to have higher femoral and lumbar BMD in the crude model (OR = 0.23; 95% CI: 0.12–0.45 and OR = 0.29; 95% CI: 0.15–0.55, respectively) and also in adjusted model for the femoral (OR = 0.29; 95% CI: 0.14–0.58) and lumbar (OR = 0.34; 95% CI: 0.17–0.67).

**Table 4:** Crude and multivariable-adjusted odds ratios and 95% CIs across tertile of HEI and DQI-I

Variables	HEI				DQI-I			
	T_1_	T_2_	T_3_	P_trend_	T_1_	T_2_	T_3_	P_trend_
**Femoral BMD**								
Crude Model	Ref.	0.46 (0.42, 1.39)	0.38 (0.20, 0.71)	**0.002**	Ref.	0.37 (0.19, 0.70)	0.23 (0.12, 0.45)	**<0.001**
Adjusted Model	Ref.	0.71 (0.37, 1.36)	0.40 (0.20, 0.78)	**0.009**	Ref.	0.34 (0.17, 0.67)	0.29 (0.14, 0.58)	**<0.001**
**Lumbar BMD**								
Crude Model	Ref.	0.43 (0.23, 0.79)	0.20 (0.10, 0.40)	**<0.001**	Ref.	0.38 (0.20, 0.72)	0.29 (0.15, 0.55)	**<0.001**
Adjusted Model	Ref.	0.38 (0.19, 0.73)	0.20 (0.10, 0.41)	**<0.001**	Ref.	0.34 (0.18, 0.67)	0.34 (0.17, 0.67)	**0.001**

BMD, bone mass density; HEI, Healthy Eating Index; DQI-I, Dietary Quality Index-International.

Adjusted for age, BMI, income, education, physical activity, taking calcium and vitamin D supplements.

These values are odds ratio (95% CIs).

Obtained from logistic regression

## Discussion

Osteoporosis is an age-related chronic condition that is a concern globally as life expectancy increases. It is agreed that lifestyle modification, mostly following high-quality dietary patterns, is the primary practical strategy to attenuate the risk of osteoporosis. As available evidence shows, few studies have illustrated correlations between HEI and DQI with BMD. The result of this case-control study among 131 postmenopausal women with osteoporosis and 131 healthy postmenopausal control group demonstrated a strong direct associations between HEI and DQI with bone health status.

Controversial results were obtained from previous studies. While some found a significant correlation between healthy eating patterns and bone health, other studies failed to find a clear association. For instance, a similar cross-sectional study among adult Iranian women revealed positive correlations between HEI and BMD at the femoral neck and lumbar spine [27]. Moreover, in a case-control study of patients with hip fracture, diets with higher HEI, DQI, the Alternate HEI (AHEI), and alternate Mediterranean Diet (aMED) score [28] were associated with a reduced risk of hip fracture [28]. Inconsistently, in a prospective cohort study among postmenopausal women, higher aMED index was correlated with a lower risk of hip fracture, and no significant relationship was seen between HEI-2010, AHEI-2010, or Dietary Approaches to Stop Hypertension (DASH) diet and the risk of hip fracture [29]. Furthermore, despite the negative relationship between dairy intake and urinary N-telopeptides/creatinine (uNTx/Cr) -as a marker of bone resorption- HEI-2005 wasn’t associated with uNTx/Cr among postmenopausal women [30]. It can be mentioned that menopausal status exerts detrimental effects on bones and thus might attenuate the protective roles of healthy dietary patterns [31].

From the standpoint of single nutrients’ effect on BMD, components like protein, fiber, vitamins A, B6, B12, C, and minerals, including sodium, calcium, magnesium, iron, zinc, and copper, can partly explicate differences in BMD score across tertiles of HEI through various mechanisms and pathways. Based on the studies, BMD can be affected by micro-and macronutrients. It has been shown that people who consumed less vegetables, fruits, and dairy products had a lower BMD [32]. Also, the occurrence of some diseases such as the outbreak of COVID-19 may have adverse effects on bone health by creating unhealthy dietary patterns [32].

In the current study, increased consumption of vegetables, fruits, legumes, whole grains, and dairy products was observed among study participants across the tertiles of HEI and DQI. It can be hypothesized that a higher intake of mentioned food groups might be the proposed reason for the observed significant linkage between HEI and DQI with BMD. Previous research has presented that adequate consumption of food groups positively influences bone health status, as well [33–35]. The beneficial effects of these groups are attributed to their nutrients, such as vitamins, minerals, protein, and fiber. For instance, protein has potential roles in modulating bone metabolism. Despite increasing calciuria, dietary protein promotes osteoblast activity and calcium absorption, which results in bone mineralization and can strengthen muscles as a protection for the skeleton [36–38]. In addition, dietary fiber intake is reported to be effective against bone loss, probably through prebiotic properties, which can increase the production of short-chain fatty acids by modulating gut microbiota and, after that, improving calcium absorption [39]. Moreover, high salt diet consumption was reported to interrupt calcium metabolism by increasing calciuria and thus negatively alter bone calcium balance [40]. Plant-based diets, such as HEI, emphasize consuming limited amounts of sodium with less added salt and processed meat, thereby reducing the risk of osteoporosis [41]. Furthermore, another plausible explanation for the protective effect of HEI and DQI against osteoporosis is the antioxidant components of such healthy diets, notably higher vitamin C intake. A U-shape correlation is suggested between vitamin C consumption and BMD. High-dose vitamin C leads to oxidative stress and cell death, and its deficiency increases osteoclast and, subsequently, decreases bone formation [42].

Some limitations of the present study can be discussed. First, due to the case-control design of the study, the causality may not be indicated clearly. Additionally, bone health status is influenced by environmental and dietary factors from birth. On the other hand, assessing dietary intake by FFQ is limited to one year. Thus, evaluating the correlation between BMD and dietary patterns in longer durations is suggested. Third, while a validated FFQ was used to evaluate the score of dietary patterns, it can be influenced by the memory of the participant, so assessment errors might happen. Moreover, since the study was conducted in Isfahan city, the result of the current study cannot be generalized to other populations. Furthermore, measuring serum biomarkers of bone turnover could be helpful in future research. Nevertheless, the present study was the first to demonstrate the association between HEI, DQI, and BMD in Isfahan, Iran. Different confounders were considered to reduce the risk of bias during the assessment. Limiting the study population to out-patient postmenopausal women attenuated the confounding effects of menopausal status and restricted low-quality diets of the care centers.

In conclusion, the result of the current study supported the hypothesis that high-quality diets with healthy patterns can clinically be effective in maintaining bone health. Therefore, recommendations regarding the consumption of nutrient-rich food groups in a healthy diet can serve as a practical non-pharmacological strategy against osteoporosis.

## Data Availability

The datasets used and/or analysed during the current study are available from the corresponding author on reasonable request.
